# Evaluation of reliability generalization of Conner-Davison Resilience Scale (CD-RISC-10 and CD-RISC-25): A Meta-analysis

**DOI:** 10.1371/journal.pone.0297913

**Published:** 2024-11-22

**Authors:** Ajele Kenni Wojujutari, Erhabor Sunday Idemudia, Lawrence Ejike Ugwu

**Affiliations:** Faculty of Humanities, North-West University, Potchefstroom, South Africa; Qatar University College of Nursing, QATAR

## Abstract

**Background:**

Resilience, a critical multi-faceted construct in psychological research, is often measured using Conner-Davison Resilience Scale (CD-RISC-10 and CD-RISC-25). This reliability generalization (RG) meta-analysis delves into evaluate the level of reliability generalization estimate of both CD-RISC-10 and CD-RISC-25 in assessing resilience across diverse populations and settings.

**Methods:**

A reliability generalization meta-analysis on the psychometric properties of CD-RISC-10 and CD-RISC-25 was conducted, encompassing 27 studies. The original versions’ psychometric properties were systematically retrieved from databases including PubMed, PsycINFO, Google Scholar, Web of Science, Science Direct, and Scopus, with a focus on studies published between 2018 and 2023. The study protocol, including the specific methods for the reliability generalization meta-analysis, was pre-registered in the Prospero database (registration number CRD42023479052). This pre-registration ensures transparency and minimizes the risk of bias in the study design and analysis.

**Results:**

The analysis revealed a combined estimated overall estimate of Cronbach’s Alpha of 0.89 (95% CI [0.87, 0.91], z = 77.20, p < 0.05), indicating a high level of reliability for CD-RISC-10 and CD-RISC-25. CD-RISC-10 exhibited an overall estimate of Cronbach’s Alpha of 0.8732 (95% CI [0.85, 0.10], z = 69.81, p < 0.05), indicating a high level of reliability, while CD-RISC-25 also demonstrated an overall estimate of Cronbach’s Alpha of 0.8922 (95% CI [0.87, 0.91], z = 77.20, p < 0.001), indicating a high level of reliability. Furthermore, CD-RISC-10 displayed commendable reliability (ωα_+_ = 0.86), slightly lower compared to the impressive reliability of CD-RISC-25 (ωα_+_ = 0.89), with a significant difference (t = 0.1159, p > 0.001). The mixed-effects model revealed a non-significant moderating effect of the CD-RISC language version on reliability estimates (coefficient = -0.0017, p <0.05).

**Conclusion:**

The results affirm the high overall reliability of both CD-RISC-10 and CD-RISC-25, with CD-RISC-25 exhibiting a slightly superior level. The non-significant moderating effect of language version suggests that the psychometric properties of these scales remain robust across different linguistic adaptations. These findings enhance our understanding of the CD-RISC scales, providing practitioners, researchers, and clinicians valuable insights for informed scale selection in diverse contexts. The commendable reliability of both scales underscores their utility in assessing and promoting resilience across varied populations and settings. Future research should explore specific contexts, demographics, and applications, enhancing their utility for diverse populations and settings.

## Introduction

Resilience, a multifaceted psychological construct reflecting an individual’s capacity to adapt and rebound in adversity, holds immense significance in research and clinical settings [1]. As resilience remains a focal point in psychological research, the CD-RISC-10 and CD-RISC-25 versions serve as indispensable instruments, providing a valuable understanding of individuals’ adaptive capacities in various contexts. The Connor-Davidson Resilience Scale, available in both 10-item (CD-RISC-10) and 25-item (CD-RISC-25) versions, has emerged as a prominent and efficient tool for assessing resilience, gaining prominence since its introduction by Connor and Davidson in 2003.

The Connor-Davidson Resilience Scale (CD-RISC) is a widely used instrument to measure an individual’s resilience, which refers to the ability to adapt and bounce back from stress, trauma, and adversity. Originally introduced in 2003 by Kathryn M. Connor and Jonathan R. T. Davidson, the CD-RISC has become a valuable instrument for psychologists, researchers, and clinicians to understand how individuals cope with stressors and traumatic experiences. The CD-RISC 10-item version is a subset of the original 25-item CD-RISC-25. Respondents rated each item on a 5-point Likert scale, ranging from 0 (‘not true at all’) to 4 (‘true nearly all the time’). The total score ranges from 0 to 40, with a higher score indicating greater psychological resilience [1,2]. This unidimensional version exhibits excellent psychometric properties, and the longer CD-RISC-10 has demonstrated cross-cultural applicability, being extensively used in mental health studies [3–6].

The Connor-Davidson Resilience Scale (CD-RISC-25) is a self-administered scale comprising 25 items, demonstrating robust psychometric properties [7,8]. Respondents rate items on a 5-point Likert scale from “not true at all” (0) to “true nearly all of the time” [9], with higher scores indicating greater resilience. The total scores range from 0 to 100 [7]. Widely employed in general population studies, clinical practice, and research, the CD-RISC-25 proves valuable in assessing resilience’s multidimensional nature across diverse populations.

Validation studies across diverse populations conducted by Skaldere-Darmudasa and Sudraba [8], Neyer et al. [10], Fekih-Romdhane et al. [11], and Song and Kang [12] have consistently demonstrated commendable psychometric properties for both CD-RISC-10 and CD-RISC-25. These studies have shown that the CD-RISC has good psychometric properties, including high internal consistency and test-retest reliability. It has also demonstrated convergent validity with other measures of psychological functioning. Therefore, it is imperative to systematically evaluate the reliability generalization (RG) of the Connor-Davidson Resilience Scale 10-item and 25-item versions (CD-RISC-10 and CD-RISC-25), given their widespread application across diverse studies and populations.

Resilience is the dynamic ability to adapt and recover from adversity, encompassing a mental and emotional fortitude that evolves across various life stages, could be influenced by diverse socio-cultural factors of an individual shaping coping mechanisms and support systems. resilience is positively associated with psychological well-being and negatively associated with psychological distress, depression, and anxiety [13,14]. Resilience can be viewed as a defence mechanism that enables people to thrive in the face of adversity and improve mental [15,16]. The relationship between resilience and mental health has a medium effect size [14]. Studies highlight a meta-analysis linking problematic Internet use and lower resilience, revealing a significant negative relationship [17,18]. The findings imply resilience potential implications for mental health and well-being.

Reliability generalization (RG) is a meta-analytic method employed to scrutinize the variability in reliability estimates of a specific test across different implementations. This approach aims to ascertain the overall trustworthiness of test results and explore the impact of sample characteristics and variability [19]. Therefore, RG involves systematic meta-analysis process, aggregating psychometric properties and sample sizes from various studies, to ascertain the general trustworthiness of a psychological assessments.

Reliability generalization (RG) emerges as a crucial meta-analytic technique for evaluating the reliability of measurement tools across diverse populations, a utility well-demonstrated by its application to various scales, including the Computational Thinking Scale [20], the WIHIC questionnaire [21], the Life Satisfaction Scale [22], the internet gaming disorder Scale (IGDS) [23], and the mother-to-Infant Bonding Scale (MIBS) [24]. Extending this perspective to the Connor-Davidson Resilience Scale (CD-RISC) scales, which have seen widespread use in various contexts, enhances the rationale for employing RG. Also, applying RG to the widely used CD-RISC scales is crucial, systematically consolidating reliability estimates to enhance arguments for their consistency and generalizability across diverse populations and research contexts.

Recent studies have consistently reported high internal consistency, good test-retest reliability, and validity for both CD-RISC-10 and CD-RISC-25 across diverse populations [25]. These include investigations into populations facing specific challenges, such as patients with Multiple Sclerosis [26,27], individuals with spinal cord injury [28] infertile patients [29], online student samples [5], healthcare professionals [30,31], vulnerable adolescents [32], university students [33], and elderly individuals [4], and Greek healthcare professionals [31]. Additionally, the effectiveness of the Spanish version of CD-RISC-10 has been affirmed in diverse contexts, including among adolescent mothers in Peru [34] and teachers [35]. Studies on the Chinese CD-RISC-10 [36] and the Arabic CD-RISC-25 [37] further give credence to the cross-cultural applicability of these resilience measures.

However, despite the wealth of recent studies demonstrating the effectiveness of CD-RISC-10 and CD-RISC-25 in measuring resilience across diverse cultural and clinical settings, there remains a concern regarding their reliability generalization. Cultural disparities, variations in measurement context, and diverse population characteristics may impact the general trustworthiness of these resilience measures. Consequently, this meta-analysis on reliability generalization (RG) aims to assess the extent of reliability generalization estimates for both CD-RISC-10 and CD-RISC-25 in evaluating resilience across diverse populations and settings.

## Methods

We conducted a reliability generalization meta-analysis (RG) to assess the psychometric properties of the Conner-Davison Resilience Scale (CD-RISC-10 and CD-RISC-25). This meta-analysis included data from 27 studies, and the review method adhered to reliability COnsensus-based Standards for the selection of health Measurement Instruments Risk of Bias Checklist (COSMIN RB) [38].

The study protocol, including the specific methods for the reliability generalization meta-analysis, was pre-registered in the Prospero database (registration number CRD42023479052, see Sup.). This pre-registration ensures transparency and minimizes the risk of bias in the study design and analysis.

### Search strategy

The systematic review followed a predefined search strategy. Databases like PubMed, PsycINFO, and Google Scholar, Web of Science, Science Direct, PLOS ONE and Scopus were queried, and relevant studies published between 2018 and 2023 were included. Identify Keywords such as Reliability, CD-RISC-10, CD-RISC-25, Conner-Davison Resilience Scale, Measurement Properties, Assessment, Psychometric properties, Internal consistency, Cronbach’s alpha, validity, and reliability. Construct Search Strings or combination: (“Reliability “OR “psychometric properties”) AND (“Conner-Davison Resilience Scale” OR “CD-RISC-10” OR “CD-RISC-25”) AND (“Cronbach’s alpha” OR “internal consistency” OR “reliability” OR “validity”)), TITLE-ABS-KEY((“Reliability generalization” OR “Measurement Properties” OR “Assessment” OR “psychometric properties”) AND (“Conner-Davison Resilience Scale” OR “CD-RISC-10” OR “CD-RISC-25”) AND (“Measurement Properties” OR “Assessment” OR “Cronbach’s alpha” OR “internal consistency” OR “reliability” OR “validity”)), TS = (“Measurement Properties” OR “Assessment” OR “Reliability generalization” OR “psychometric properties”) AND TS = (“Conner-Davison Resilience Scale” OR “Measurement Properties” OR “Assessment” OR “CD-RISC-10” OR “CD-RISC-25”) AND TS = (“Cronbach’s alpha” OR “internal consistency” OR “reliability” OR “validity”)). No language restrictions were imposed, aiming for a comprehensive inclusion of relevant studies on CD-RISC scales’ validity and reliability.

### Selection criteria

The study aims to inclusively considered research on the psychometric properties and reliability assessment of the Conner-Davison Resilience Scale (CD-RISC-10 and CD-RISC-25) across varied populations and settings, ensuring a comprehensive and representative sample. This inclusivity extends to validation, inter-rater reliability, test-retest reliability, internal consistency, and cross-cultural validation studies. The selection criteria are designed to promote diversity, given the global application of the CD-RISC scales, by encompassing studies that explore resilience in different demographic groups, cultural contexts, and geographic locations.

Exclusion criteria prioritize the relevance and reliability of the data, excluding studies that do not report on reliability or psychometric properties, publications not in English, reviews, conference abstracts, editorials, case reports, and those lacking sufficient data. This approach ensures a nuanced understanding of resilience across diverse contexts, contributing to a more representative and robust meta-analysis.

### Data extraction

The selection of studies for Reliability Generalization (RG) adhered to specific eligibility criteria, involving two independent reviewers who screened titles, authors, publication details, Digital Object Identifiers (DOIs) or URLs, and abstracts of each study. Following this initial screening, full-text articles meeting the criteria underwent a comprehensive assessment for final inclusion, with any discrepancies resolved through consultation with a third reviewer. The focus of the subsequent data extraction process was on the psychometric properties of the Conner-Davison Resilience Scales (CD-RISC-10 and CD-RISC-25). This encompassed gathering information on study characteristics, demographics, reliability coefficients, inter-rater reliability, test-retest, and validity measures. Extracting data on sample characteristics, including size and participant demographics, enabled a subtle interpretation of reliability across diverse populations, contributed to the overall synthesis of findings. The extraction process also considered potential moderators, including language version, age, gender, and cultural factors. The entire process, from study selection to data extraction, was meticulously documented for consistency and accuracy. In instances where disagreements arose, consensus was reached through collaborative discussions between reviewers, ensuring a robust and reliable foundation for the meta-analysis.

### Quality assessment

Two independent investigators conducted Quality Assessment of Diagnostic Accuracy Studies (QUADAS-2, Whiting, et al. 2021) and COnsensus-based Standards for the selection of health Measurement Instruments Risk of Bias checklist (COSMIN,) [38] to assess the quality of the include studies. We conducted a comprehensive evaluation of multiple studies using the QUADAS-2 framework, providing critical insights into the methodological quality of each study (S1 Fig in S2 File). The categories including patients’ selection, Index Text, Reference Standard, Flow and Timing, and Overall Assessment offer insight into the methodological rigor and potential biases inherent in each study. QUADAS-2 tool was used to rate each included study on a 4-point scale “Low” “Some concern”, and “High”.

The Quality Assessment of Diagnostic Accuracy Studies (QUADAS-2,) [39] and the COnsensus-based Standards for the selection of health Measurement Instruments Risk of Bias checklist (COSMIN RB,) [38] (S1 Table in S2 File). The QUADAS-2 framework, employed for diagnostic accuracy studies, allows for a detailed analysis of patients’ selection, Index Test, Reference Standard, Flow and Timing, and Overall Assessment, shedding light on methodological rigor and potential biases in each study. Utilizing the QUADAS-2 tool, each included study underwent a rigorous evaluation on a 4-point scale, categorizing the assessments as “Low,” “Some concern,” or “High.” Notably, the studies under consideration received a classification of “Low,” indicating a favourable outcome in terms of methodological quality, reinforcing their credibility and reliability in the context of the assessment criteria [39].

To assess the included studies using the COSMIN Risk of Bias (RB) checklist, attention is directed toward key aspects related to the measurement properties of the questionnaire. COSMIN RB primarily scrutinizes reliability, validity, and responsiveness, as outlined by Mokkink in 2018. The COSMIN RB checklist comprises ten checkboxes, each corresponding to specific metric properties. Each checkbox contains items addressing various aspects of design and statistical methods.

The studies included in the evaluation were systematically rated for reliability, validity, and responsiveness (S1 Table in S2 File). The classifications for these assessments are denoted as “Very Good,” “Adequate,” or “Doubtful.” These ratings provide a detailed understanding of the methodological strengths and potential limitations inherent in each study, contributing valuable insights into the reliability, validity, and responsiveness of the measurement properties under investigation. Methodological quality significantly impacts meta-analysis findings. The robust methodologies of each included study contribute to reliability and precise estimates, enhancing overall credibility, generalizability, and validity of the meta-analysis on CD-RISC scales in this study.

### Data analysis

In data analysis, we employed meta-analysis statistical methods to synthesize findings across studies. Effect sizes for validity and reliability coefficients were calculated using random-effects models, considering heterogeneity. A qualitative analysis will summarize the Conner-Davison Resilience Scale’s reliability. Additionally, a meta-analysis, employing a random-effects model, will pool reliability coefficients. Heterogeneity was explored through the I^2^ statistic and subgroup analyses. Data analysis was conducted using R Studio with the metafor package. In this meta-analysis, effect sizes were calculated based on Cronbach’s alpha, which measures the internal consistency or reliability of a scale.


$$\text{The}\;\text{formula}\;\text{for}\;\text{effect}\;\text{size}\;\text{is}\sqrt{Cronbach^{\prime }s\;alpha\;}x\;\left(1-\frac{1}{Sample\;Size}\right)$$

This approach allows for the synthesis of findings regarding the reliability of CD-RISC-10 and CD-RISC-25 across multiple studies. A random-effects model was chosen due to the likelihood of heterogeneity among studies. The random-effects model accounts for variations in true effects across different populations or contexts. This choice acknowledges that the included studies may have different underlying true effects, making the random-effects model more suitable for generalizing findings beyond the specific studies included. Heterogeneity among studies was assessed using the I^2^ statistic. I^2^ quantifies the percentage of total variation across studies due to heterogeneity rather than chance. Higher values of I^2^ indicate greater heterogeneity. Subgroup analyses were conducted to explore potential sources of heterogeneity, such as variations in study design, participant characteristics, or other factors. Addressing heterogeneity is crucial as it impacts the interpretation and generalizability of the meta-analysis results.

High heterogeneity suggests variability in reliability estimates among studies. It prompts a careful interpretation of the overall findings, recognizing that the true reliability of CD-RISC-10 and CD-RISC-25 may differ across diverse populations or under various conditions. Subgroup analyses help identify factors contributing to heterogeneity, offering insights into the sources of variation. R Studio, a comprehensive integrated development environment, was utilized for its flexibility and powerful tools. The metafor package in R Studio facilitated the meta-analysis implementation. Specific functions such as ‘**metagen()’** were employed for combining effect sizes and estimating overall effects. The ‘**forest ()’** function was used to create the forest plot, providing a visual representation of the meta-analysis results. The code structure ensures transparency and allows for further customization or adaptation based on the specific needs of the analysis.

### Ethical considerations

Due to the nature of our study, formal ethical clearance was not obtained for its execution. Nonetheless, in our diligent examination of the reliability generalization of the Conner-Davidson Resilience Scale through meta-analysis, we are committed to upholding ethical standards. This commitment involves ensuring the confidentiality of participant information, obtaining informed consent, and maintaining research integrity. Moreover, we are dedicated to transparency and methodological quality in our research, as evidenced by our adherence to PROSPERO protocol registration and the utilization of PRISMA guidelines. These practices guarantee a thorough and responsible approach to our study, instilling confidence within the scientific community regarding the validity and reliability of our findings.

## Results

### Reliability report inducement of the included studies

**Fig 1:**
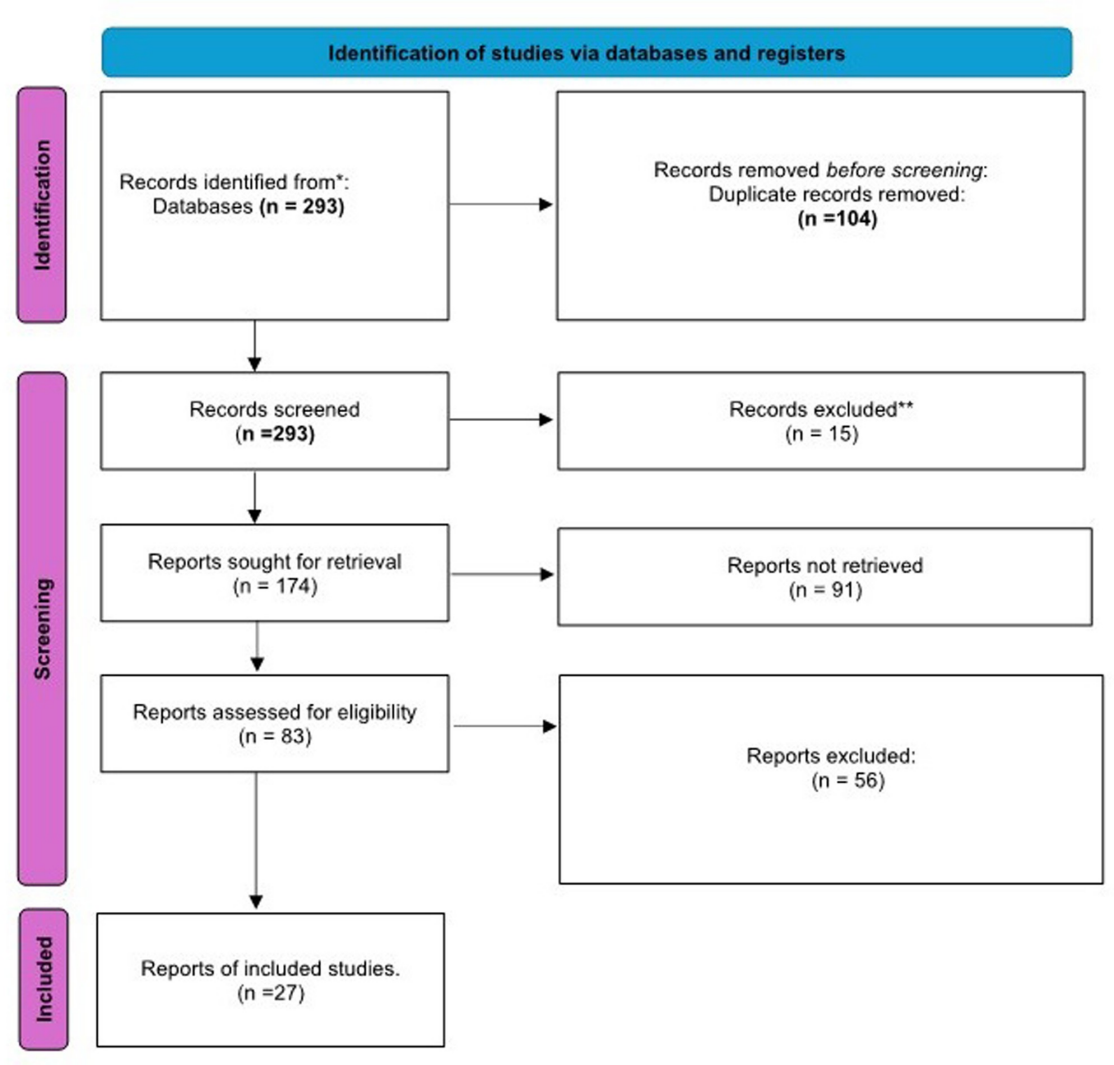
PRISMA flow chart of study selection for reliability generalization meta-analysis of CD-RISC scales.

A total of 293 research articles were identified through the search of database and other sources as illustrated in the PRISMA Flow chart (see Fig 1). Fig 1 presents these initial identified 293 research articles in the database and other source, 262 (90.66%) of the identified articles were discarded based on the study exclusion criteria, 185(64.01%) studies were duplicated, 104(35.99%) studies abstracts were screened, 89(30.80%) full articles were assessed for eligibility, while 27(78.03%) studies were included in the meta-analysis base the study inclusion criteria. The 27 studies included in reliability generalisation (RG) meta-analysis, reported the reliability coefficient (Cronbach’s Alpha) ranged from 0.82 to 0.93 for either CD-RISC-10 or CD-RISC-25. The 27 studies included in the RG, only 9(33.33%) studies were conducted on CD-RISC-25, 18(66.67%) studies were conducted on CD-RISC-10, 7(25.93%) studies used English-version, 9(33.33%) studies use Spanish-version, 2(7.41%) studies used Chinese-version and Arabic-version, while the remaining 1(3.7%) studies used French-Versions, Farsi-version, Russian-version, Greek-version, German -versions, and Swedish version.

### Reliability generalisation meta-analysis

**Fig 2:**
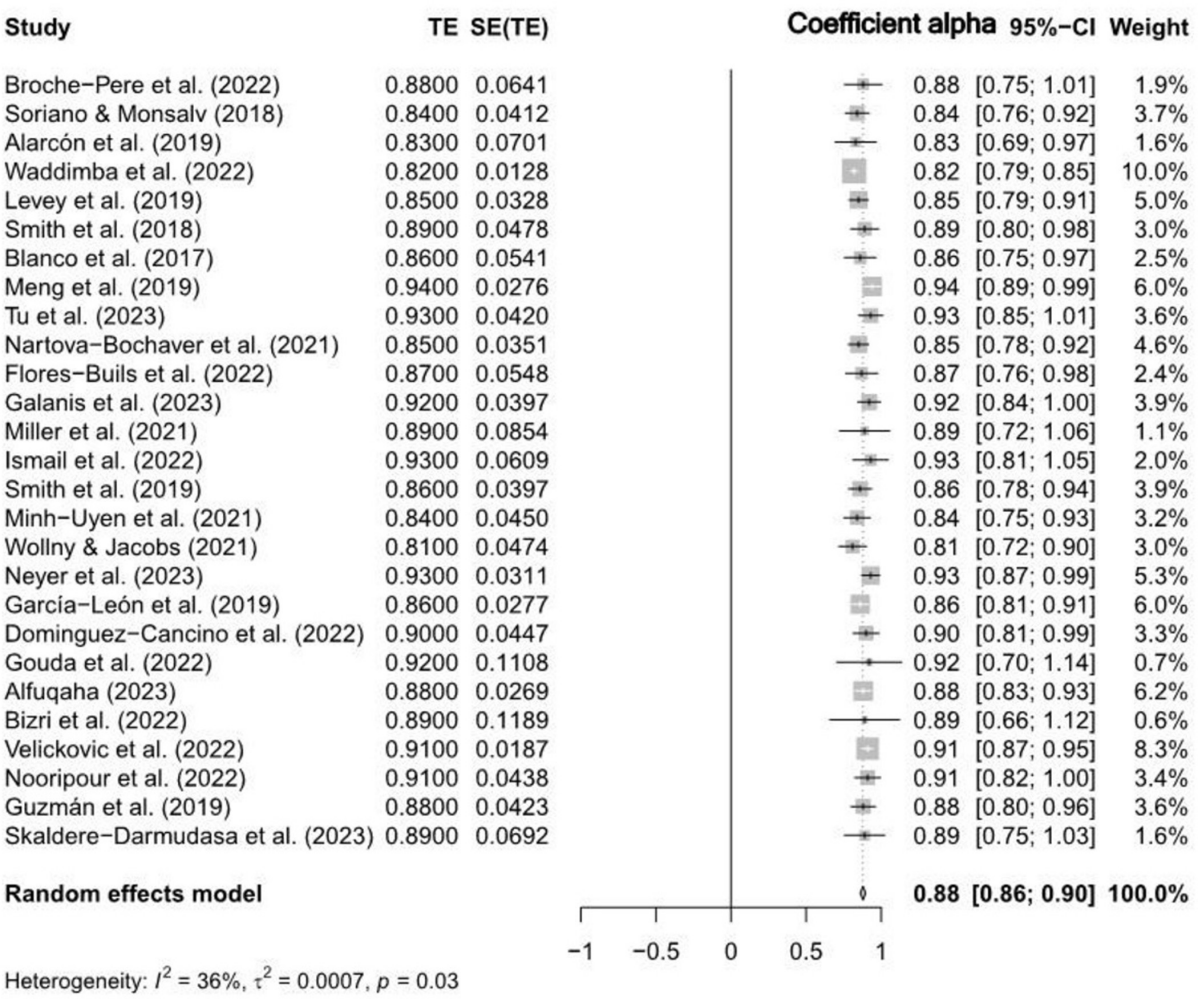
Forest plot of combined reliability estimates for CD-RISC-10 and CD-RISC-25 scales.

The RG meta-analysis was to assess the overall reliability of the CD-RISC-10 and CD-RISC-25 scales based on a comprehensive review of included studies. Table 1 results of the analysis revealed a combined estimated overall reliability of 0.88 (95% CI [0.86, 0.89]) for both scales combined as shown in the Forest Plot (see Fig 2). This finding suggests that, on average, CD-RISC-10 and CD-RISC-25 are exceptionally reliable measurement tools, providing consistent and trustworthy assessments of resilience.

**Table 1:** Summary of random-effects model of CD-RISC-10 and CD-RISC-25 reliability generalisation meta-analysis.

Total scales	k	Estimate α_+_	ωα_+_	zval	90%CL	Q	I^2^
					LL(UL)		
**Coefficient alpha**							
**Combine**	**27**	**0.88**		**92.71**	0.86(0.89) ***	40.53***	**35.9%**
CD-RISC-10	18	0.87	0.86	69.81	0.89(0.94) ***	32.22***	47.2%
CD-RISC-25	9	0.89	0.89	77.20	0.87(0.91) ***	2.80	0.0%
**Model**	**2**	**0.91**		**75.38**	0.88 (0.93) ***	**0.1870**	**0.00%**

**Note:** k = number of studies, Q = Cochran’s Heterogeneity Q statistics, I^2 =^ Heterogeneity index, ωα_+ =_ weighed mean Cronbach’s alpha.

The test for heterogeneity resulted in a highly significant outcome (Q = 40.53, df = 26, p < 0.05), confirming the existence of considerable heterogeneity among the studies. The high statistical significance (z = 92.71, p < 0.05) indicates that this estimate is robust and dependable (see Table 1). However, it is important to note the substantial heterogeneity analysis revealed a moderate level of variability among the studies. The between-study variance (τ^2^) was estimated at 0.0007 (95% CI [0.0000, 0.0011]), and the standard deviation of true effects (τ) was 0.0271 (95% CI [0.0000, 0.0326]) (see Fig 2). The I^2^ statistic suggested that 35.9% (95% CI [0.0%, 59.8%]) of the total variation could be attributed to heterogeneity, and the Higgins’ heterogeneity factor (H) was 1.25 (95% CI [1.00, 1.58]) (see Fig 2), which suggests that variations in sample characteristics, study designs, or other factors contribute to the observed differences in reliability estimates. These findings imply that CD-RISC-10 and CD-RISC-25 are reliable resilience measurement tools, but their high heterogeneity indicates significant variability in reliability estimates across the analysed studies.

**Fig 3:**
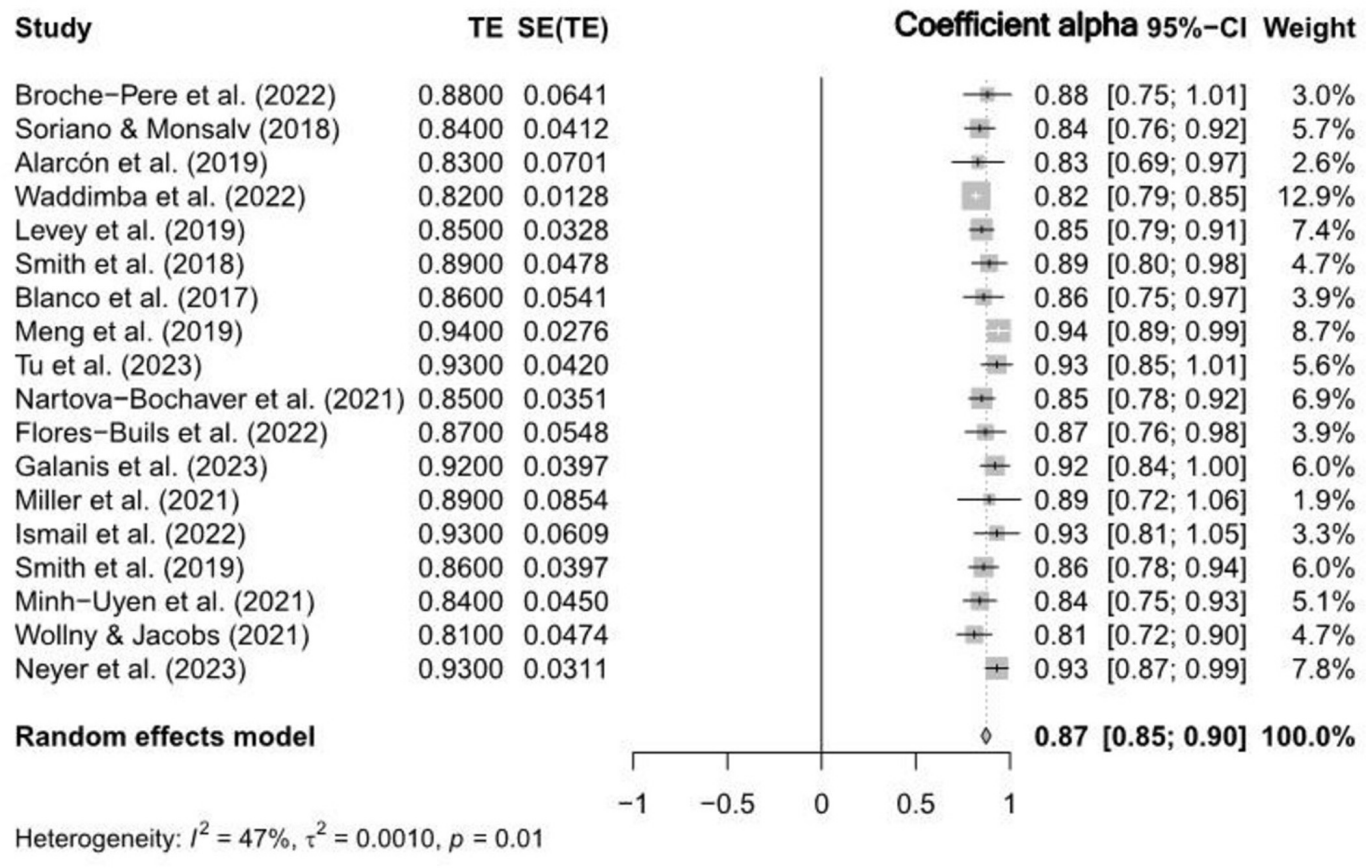
Forest plot of CD-RISC-10 reliability estimates.

**Fig 4:**
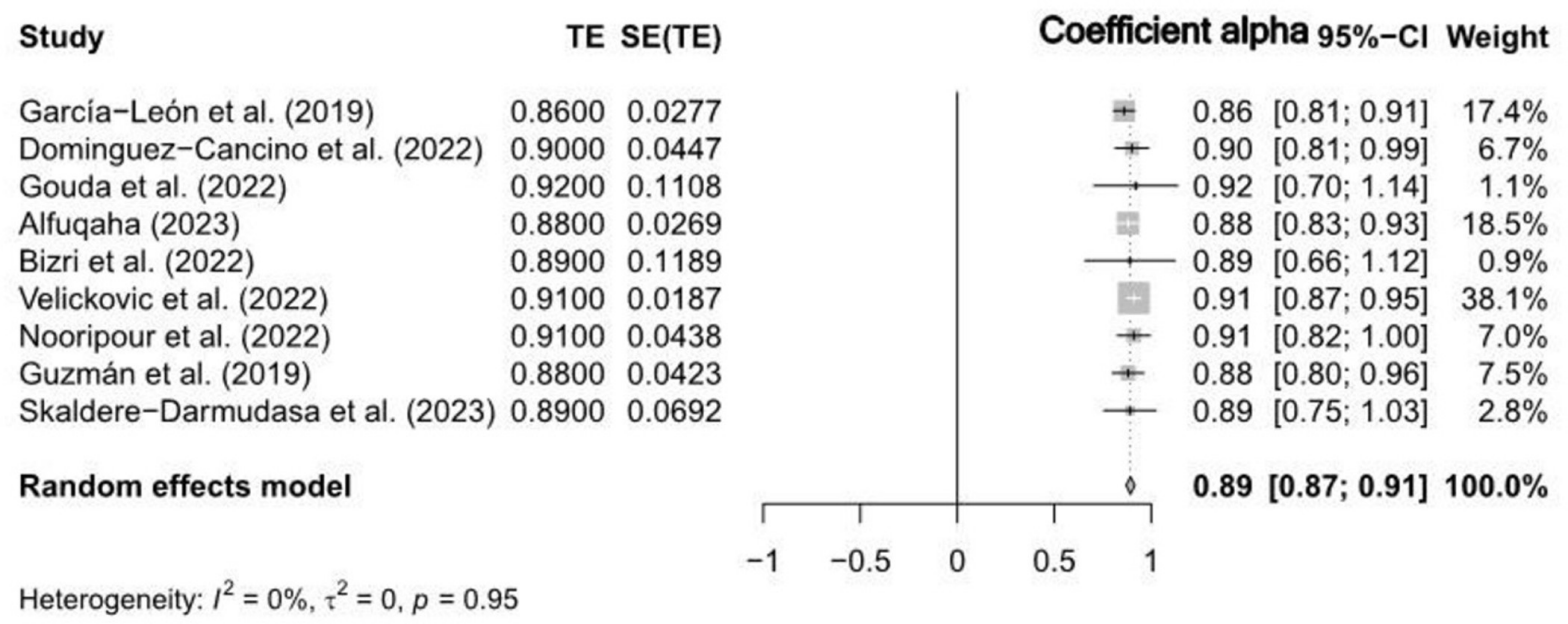
Forest plot of CD-RISC-25 reliability estimates.

The RG meta-analysis also to investigate potential differences in the reliability estimates between CD-RISC-10 and CD-RISC-25 scales, as reported in the included studies. Table 1 showed that CD-RISC-10 had an estimated reliability of 0.87 (95% CI [0.89, 0.94]) also illustrated in the Forest Plot showing weighed mean alpha coefficient (see Fig 3), while CD-RISC-25 had an estimated reliability of 0.89 (95% CI [0.87, 0.91]) illustrated in the Forest Plot showing weighed alpha coefficient (see Fig 4). These results indicate that both scales exhibit strong reliability; however, there is a slight difference favouring CD-RISC-10 in terms of reliability.

**Fig 5:**
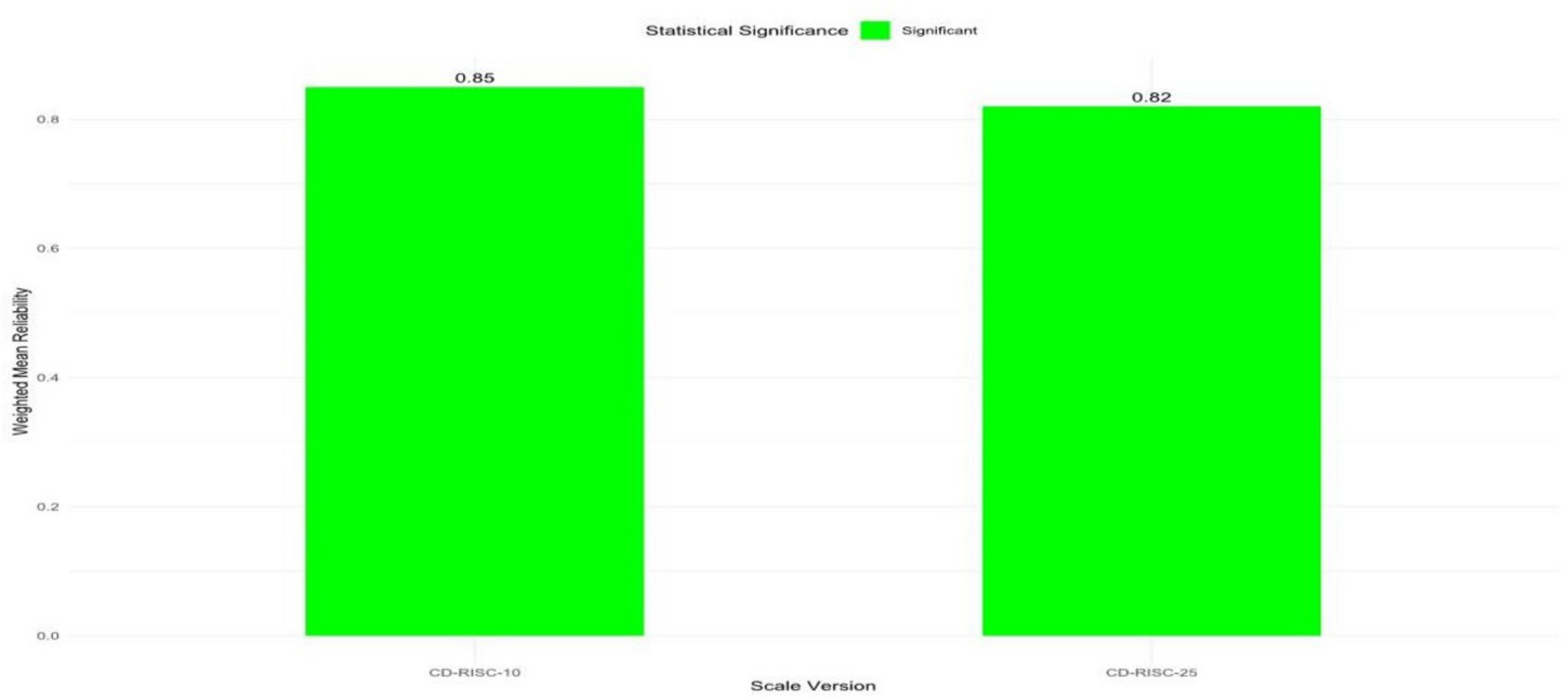
GGPlot2 weighted alpha coefficient difference between CD-RISC-10 and CD-RISC-25.

Table 1 results also showed a commendably (ωα_+_ = 0.86) for CD-RISC-10 that is slightly lower, compared to CD-RISC-25 with impressive level of reliability (ωα_+_ = 0.89), (*t* = 0.1159, p > 0.05). this signifying no statistically significant difference in reliability estimates between CD-RISC-10 and CD-RISC-25 in the selected studies as shown in the ggplot2 weighed alpha coefficient difference (see Fig 5).

Table 1 result showed the statistical testing further supported the similarity in reliability between CD-RISC-10 and CD-RISC-25, with no significant difference observed (z = 0.874, p = 0.382). Additionally, heterogeneity was low for CD-RISC-25 (I^2^ = 0.00%) but higher for CD-RISC-10 (I^2^ = 91.36%).

The CD-RISC-10 test for heterogeneity resulted in a highly significant outcome (Q = 32.22, df = 17, p < 0.05), confirming the existence of considerable heterogeneity among the studies. The high statistical significance (z = 69.81, p < 0.05) indicates that this estimate is robust and dependable (see Table 1). The further CD-RISC-10 test for heterogeneity analysis revealed moderate variability among the studies. The between-study variance (τ^2^) was estimated as 0.0010 (95% CI [0.0001, 0.0023]), with a corresponding standard deviation of true effects (τ) of 0.0323 (95% CI [0.0078, 0.0483]). The I^2^ statistic indicated 47.2% (95% CI [8.7%, 69.5%]) of the total variation could be attributed to heterogeneity, and the Higgins’ heterogeneity factor (H) was 1.38 (95% CI [1.05, 1.81]) (**see Fig 3**), highlighting the presence of considerable variability in the research landscape.

In contrast, The CD-RISC-25 test for heterogeneity resulted not statistically significant (Q = 2.80, df = 8, p > 0.05), suggesting homogeneity in effect sizes among the included studies. The high statistical significance (z = 77.20, p < 0.05), indicating a high level of reliability (see Table 2). The further CD-RISC-25 test for heterogeneity analysis revealed minimal variability among the studies. The between-study variance (τ^2^) was estimated as 0 (95% CI [0.0000, 0.0002]), with a corresponding standard deviation of true effects (τ) of 0 (95% CI [0.0000, 0.0124]). The I^2^ statistic indicated no significant heterogeneity (0.0%, 95% CI [0.0%, 64.8%]), and the Higgins’ heterogeneity factor (H) was 1.00 (95% CI [1.00, 1.69]) (see Fig 4), highlighting the presence of considerable variability in the research landscape. These findings imply that that CD-RISC-10 and CD-RISC-25 have strong reliability, with CD-RISC-10 having a slight advantage. However, no significant difference was found within the selected studies. CD-RISC-10 showed greater heterogeneity in reliability estimates.

**Table 2:** Summary of mixed-effects model meta-analysis of CD-RISC-10 and CD-RISC-25 of reliability generalisation by scales language version (k = 27).

Moderator	Model				Heterogeneity			
	Estimate	SE	z	95%CL	Q	QM	I^2^	R^2^
Intercept (intrcpt)	-0.1186	0.0226	-5.25***	-0.16, (-0.07)				
Scale Language-Version	-0.0017	0.0031	-0.54	-0.01, (0.01)	44.77***	0.29	44.16%	0.00%

**Note:** k = number of studies, Q = Cochran’s Heterogeneity Q statistics, I^2 =^ Heterogeneity index, QM = moderation Heterogeneity QM statistics.

### Analysis of moderator variables

**Fig 6:**
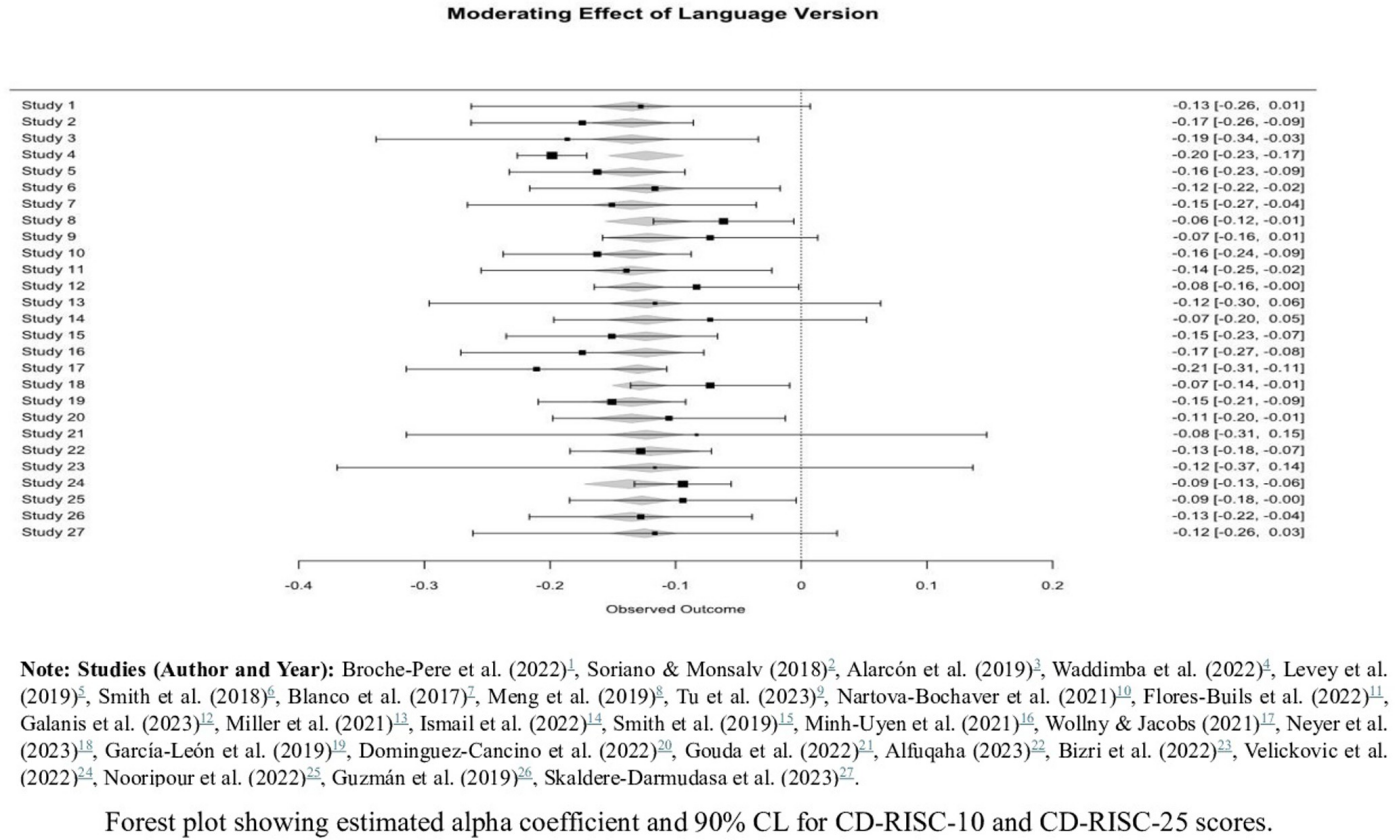
Moderating effect of language version on CD-RISC reliability estimates.

The RG meta-analysis objective final objective to evaluate the moderating effect of the language version of CD-RISC-10 and CD-RISC-25 reliability estimates in the included studies. Table 2 results of the mixed-effects model show non-significant moderating effect of the CD-RISC language version reliability estimates (coefficient = -0.0017, p = 0.5903). This suggests that, on average, the language version used to assess CD-RISC-10 and CD-RISC-25 did not significantly impact the reliability of these scales across studies (**see Fig 6**). The results show overall variability in reliability estimates was modestly attributed to the language version (R^2^ = 0.00%), there was a considerable level of residual heterogeneity (I^2^ = 44.16%). Also, the test for heterogeneity for CD-RISC-10 and CD-RISC-25 was significant (QM (df = 1) = 0.29, p = 0.5903), This residual heterogeneity indicates that other unaccounted factors, beyond language version, may contribute to variations in reliability. Overall, these findings support the cross-cultural utility and robustness of CD-RISC-10 and CD-RISC-25 as reliable measures of resilience, regardless of the language version used.

## Discussion

The overarching goal of this study encompassed three key objectives: first, to assess the overall reliability estimates of the CD-RISC-10 and CD-RISC-25 scales; second, to examine potential differences in the reliability estimates between these two scales; and third, to evaluate the moderating effect of language versions on the reliability estimates of both scales.

The current study aligns with previous research, providing robust support for the reliability, comparability, and cross-cultural applicability of the CD-RISC-10 and CD-RISC-25 scales. The findings reaffirm the high internal consistency, robust test-retest reliability, and validity of both scales, consistent with previous studies [24,26–28]. The study results suggest a degree of methodological consistency in resilience measurement across different contexts. The uniformity in outcomes across diverse demographic groups enhances the generalizability of the findings.

Additionally, our outcomes align with the recent study by Zhang et al. [28], reinforcing the robustness of the CD-RISC-10 and CD-RISC-25 scales. Zhang et al. [29] found good internal consistency and convergent validity in individuals with spinal cord injuries, mirroring our own findings and suggesting the scales’ applicability across diverse populations facing distinct challenges. Despite the overall alignment with existing literature, variations in sample characteristics, data collection methods, and statistical analyses might contribute to discrepancies in specific reliability estimates. Differences in the cultural adaptation and translation processes of the scales across languages may introduce variations in understanding and response patterns.

The second aim of this study was to scrutinize potential variations in the reliability estimates between the CD-RISC-10 and CD-RISC-25 scales. Our results reveal robust reliability for both scales, suggesting that they perform comparably in assessing resilience. This aligns with the findings of Zhang [29], who, in their research on infertile patients, observed high internal consistency and demonstrated convergent/divergent validity, affirming the reliability and validity of both CD-RISC-10 and CD-RISC-25. The consistency of these outcomes with our study bolsters the argument for the reliability of both scales across diverse contexts.

Moreover, our results resonate with the work of Wollny and Jacobs [5], who found good reliability and validity of the CD-RISC-10 among an online student sample. The convergence of our findings with this study underscores the consistent performance of the CD-RISC-10 in different populations, further emphasizing its robustness as a tool for measuring resilience.

Additionally, our study aligns with the research conducted by Elkudssiah Ismail et al. [30], which attested to the validity and reliability of the CD-RISC-10 in measuring resilience among healthcare professionals in Malaysia. The consistent validation of the CD-RISC-10 in diverse occupational contexts adds to the growing body of evidence supporting its applicability beyond specific demographic groups.

The final objective of this study aimed to assess the moderating effect of the language version on the reliability estimates of CD-RISC-10 and CD-RISC-25. The results revealed a non-significant moderating effect of the CD-RISC language version on reliability estimates. This aligns with the research conducted by Lfuqaha [37], which demonstrated strong evidence of validity and reliability for the Arabic version of CD-RISC-25, establishing it as a valuable tool for measuring resilience in Arabic-speaking populations. The concordance of our findings with this study suggests that the reliability of the CD-RISC scales transcends language variations, making them versatile instruments across different linguistic contexts.

Our results are consistent with the study by Galanis [31], who confirmed the excellent reliability of the Greek language version of CD-RISC-10 among nurses, affirming its validity for resilience assessment within Greece’s healthcare system. This consistency underscores the adaptability and reliability of the CD-RISC scales across diverse linguistic frameworks.

Furthermore, our findings are in harmony with the study by Bizri et al. [40], which found high reliability and validity of the Arabic CD-RISC-25, emphasizing its appropriateness as a tool for assessing resilience among Lebanese women. This agreement reinforces the notion that language variations do not compromise the reliability of the CD-RISC scales.

The study by Flores-Buils et al. [35] also aligns with our results, validating the Spanish version of CD-RISC-10 for assessing teacher resilience and confirming its high reliability. This supports the utility of the CD-RISC scales in educational research and training program assessments across different language contexts.

Moreover, our findings correspond with the research by Minh-Uyen and Im [33], affirming the reliability and accuracy of the CD-RISC-10 in the Vietnamese language-version for evaluating resilience levels among university students. The adaptability of the CD-RISC scales to different languages is crucial for their widespread applicability in diverse cultural and linguistic settings.

Finally, Velickovic et al. [41] found the Swedish version of CD-RISC-25 to be a reliable and valid tool for assessing psychological resilience. This conclusion aligns with our review results, further supporting the notion that CD-RISC scales maintain their reliability across various language versions. The collective evidence presented in this study emphasizes the cross-cultural robustness of the CD-RISC scales, making them valuable instruments for resilience assessment on a global scale.

This comprehensive study not only reaffirms the robustness of CD-RISC-10 and CD-RISC-25 in assessing resilience but also highlights their consistent performance across diverse populations and languages. These findings contribute valuable insights to the field of resilience research and emphasize the broad applicability of these scales in both clinical and research settings worldwide.

### Implications

The study makes a substantial contribution to resilience research, reinforcing the reliability and versatility of CD-RISC-10 and CD-RISC-25 scales across diverse populations. The observed high internal consistency and robust test-retest reliability align with existing resilience theories, providing a stable foundation for further theoretical development. Additionally, confirming the comparability of reliability estimates between both scales contributes to the ongoing discourse on scale selection, offering practical flexibility for researchers.

The global applicability of CD-RISC-10 and CD-RISC-25 across languages and cultures holds significant practical implications, enabling mental health professionals, educators, and researchers to confidently use these instruments in diverse linguistic contexts. Moreover, the study supports the utility of CD-RISC scales in specific demographic groups, showcasing their effectiveness in capturing resilience levels among individuals with Multiple Sclerosis, spinal cord injuries, healthcare professionals, nurses, teachers, and university students in various occupational and health-related contexts.

### Limitation and further research

The consistent replication of high internal consistency, test-retest reliability, and validity across various studies and populations underscores the robustness and generalizability of the CD-RISC-10 and CD-RISC-25 scales as reliable tools for assessing resilience. However, it is crucial to acknowledge potential limitations, such as the variability in sample sizes, exclusion of non-English articles, and demographic characteristics across studies, which should be considered in the broader context of resilience research. Further exploration and critical evaluation of these scales in varied settings will contribute to a more comprehensive understanding of their utility and potential limitations.

Future research should delve into more specific contexts to comprehensively understand the performance of the CD-RISC scales. This entails assessing their reliability in particular clinical populations, cultural environments, or age brackets, thereby improving the precision of their application. A thorough investigation into how demographic factors impact the reliability of CD-RISC-10 and CD-RISC-25 is warranted to refine their applicability across diverse populations. This involves scrutinizing potential age, gender, or cultural variations in resilience measurement. Additionally, there is a pressing need for validation studies in populations that are currently overlooked or underrepresented in resilience research, including diverse cultural and ethnic groups, as well as populations confronting unique challenges.

## Conclusion

The results affirm the high overall reliability of both CD-RISC-10 and CD-RISC-25, with CD-RISC-25 exhibiting a slightly superior level. The non-significant moderating effect of language version suggests that the psychometric properties of these scales remain robust across different linguistic adaptations. These findings enhance our understanding of the CD-RISC scales, providing practitioners, researchers, and clinicians valuable insights for informed scale selection in diverse contexts. The commendable reliability of both scales underscores their utility in assessing and promoting resilience across varied populations and settings. Future research should explore specific contexts, demographics, and applications, enhancing their utility for diverse populations and settings.

## Supporting information

S1 Checklist PRISMA 2020 checklist. (DOCX)

S1 File (PDF)

S2 File (DOCX)
