# Supplementary material for: Evaluation of reliability generalization of Conner-Davison Resilience Scale (CD-RISC-10 and CD-RISC-25): A Meta-analysis
Source: PLoS One. 2024 Nov 22;19(11):e0297913. doi: 10.1371/journal.pone.0297913 (PMC11584089; doi:10.1371/journal.pone.0297913)
Supplement: S2 File — (DOCX) [file pone.0297913.s003.docx]

**Supplemental information**


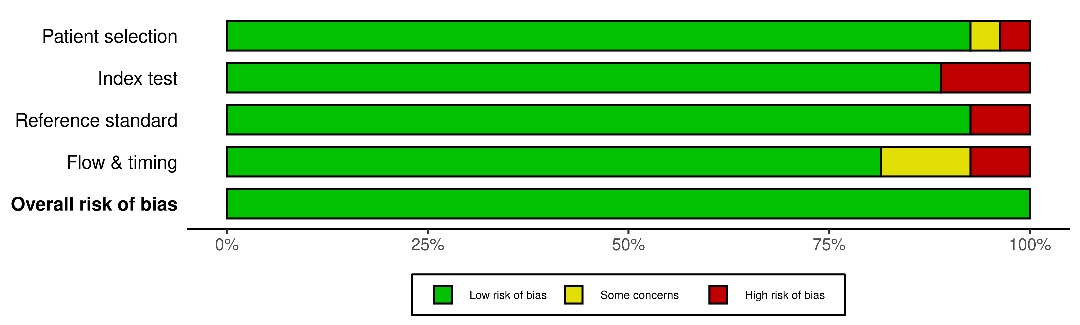


**Fig. 1:** QUADAS-2 Assessments for Included Studies

**Table 1:** Summary of COSMIN Risk of Bias (RB) Checklist Assessments for Included Studies

| **Author (Year)** | **Sample Size** | **Reliability** | **Validity** | **Responsiveness** |
| --- | --- | --- | --- | --- |
| Broche-Pere (2022) | 214 | Adequate | Very Good | Adequate |
| Soriano & Monsalv (2018) | 494 | Adequate | Adequate | Adequate |
| Alarcón (2019) | 169 | Adequate | Doubtful | Adequate |
| Waddimba (2022) | 5023 | Adequate | Adequate | Adequate |
| Levey (2019) | 789 | Adequate | Doubtful | Adequate |
| Smith (2018) | 390 | Very Good | Adequate | Adequate |
| Blanco (2017) | 294 | Adequate | Adequate | Adequate |
| Meng (2019) | 1238 | Very Good | Adequate | Adequate |
| Tu (2023) | 528 | Very Good | Adequate | Adequate |
| Nartova-Bochaver (2021) | 689 | Adequate | Adequate | Adequate |
| Flores-Buils (2022) | 290 | Adequate | Adequate | Adequate |
| Galanis (2023) | 583 | Adequate | Adequate | Adequate |
| Miller (2021) | 122 | Adequate | Adequate | Adequate |
| Ismail (2022) | 251 | Very Good | Adequate | Adequate |
| Smith (2019) | 546 | Adequate | Adequate | Adequate |
| Minh-Uyen (2021) | 414 | Adequate | Adequate | Adequate |
| Wollny & Jacobs (2021) | 360 | Adequate | Adequate | Adequate |
| Neyer (2023) | 962 | Adequate | Adequate | Adequate |
| García-León (2019) | 1119 | Adequate | Adequate | Adequate |
| Dominguez-Cancino (2022) | 451 | Adequate | Adequate | Adequate |
| Gouda (2022) | 75 | Adequate | Adequate | Adequate |
| Alfuqaha (2023) | 1220 | Adequate | Adequate | Adequate |
| Bizri (2022) | 63 | Adequate | Adequate | Adequate |
| Velickovic (2022) | 2599 | Adequate | Adequate | Adequate |
| Nooripour (2022) | 475 | Adequate | Adequate | Adequate |
| Guzmán (2019) | 492 | Adequate | Adequate | Adequate |
| Skaldere-Darmudasa (2023) | 186 | Adequate | Adequate | Adequate |
